# A pH-activatable and aniline-substituted photosensitizer for near-infrared cancer theranostics†
†Electronic supplementary information (ESI) available: Experimental details and supplementary figures. See DOI: 10.1039/c5sc01721a


**DOI:** 10.1039/c5sc01721a

**Published:** 2015-07-13

**Authors:** Jiangwei Tian, Jinfeng Zhou, Zhen Shen, Lin Ding, Jun-Sheng Yu, Huangxian Ju

**Affiliations:** a State Key Laboratory of Analytical Chemistry for Life Science , State Key Laboratory of Coordination Chemistry , School of Chemistry and Chemical Engineering , Nanjing University , Nanjing 210093 , P. R. China . Email: hxju@nju.edu.cn ; Fax: +86 25 83593593 ; Tel: +86 25 83593593

## Abstract

This work reports a newly designed pH-activatable and aniline-substituted aza-boron-dipyrromethene as a trifunctional photosensitizer to achieve highly selective tumor imaging, efficient photodynamic therapy (PDT) and therapeutic self-monitoring through encapsulation in a cRGD-functionalized nanomicelle. The diethylaminophenyl is introduced in to the structure for pH-activatable near-infrared fluorescence and singlet oxygen (^1^O_2_) generation, and bromophenyl is imported to increase the ^1^O_2_ generation efficiency upon pH activation by virtue of its heavy atom effect. After encapsulation, the nanoprobe can target α_v_β_3_ integrin-rich tumor cells *via* cRGD and is activated by physiologically acidic pH for cancer discrimination and PDT. The fascinating advantage of the nanoprobe is near-infrared implementation beyond 800 nm, which significantly improves the imaging sensitivity and increases the penetration depth of the PDT. By monitoring the fluorescence decrease in the tumor region after PDT, the therapeutic efficacy is demonstrated *in situ* and in real time, which provides a valuable and convenient self-feedback function for PDT efficacy tracking. Therefore, this rationally designed and carefully engineered nanoprobe offers a new paradigm for precise tumor theranostics and may provide novel opportunities for future clinical cancer treatment.

## Abstract 2

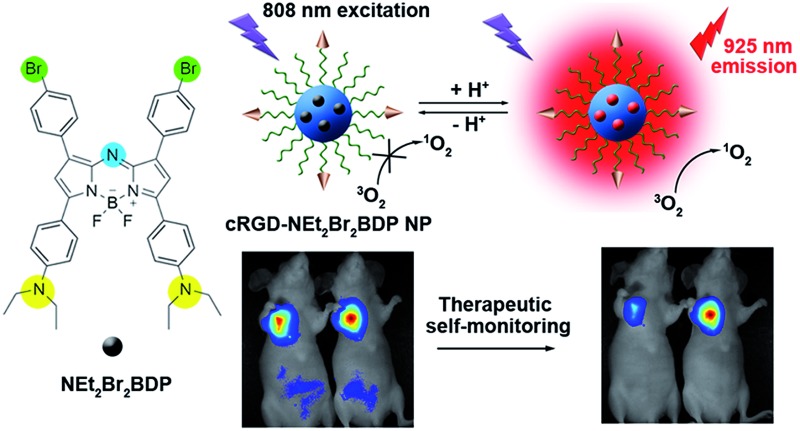
A trifunctional photosensitizer was designed to achieve highly selective near-infrared tumor imaging, efficient photodynamic therapy and therapeutic self-monitoring.

## Introduction

In current cancer research, the design of a theranostic agent that combines diagnosis and therapy into a single platform offers exciting prospects for the development of personalized medicine.^1^ Many well established photosensitizers of the first, second and third generation^2*a*^ have been used for the construction of theranostic agents.^2^ These are generally nontoxic in their native state, however, under irradiation with the appropriate wavelength and power, they can transfer the absorbed photon energy to the surrounding molecular oxygen (^3^O_2_), generating cytotoxic singlet oxygen (^1^O_2_) to kill cancer cells.^3^ This technology is well known as photodynamic therapy (PDT) and has been largely used for the treatment of many localized superficial cancers.^3^ Meanwhile, upon light excitation, the photosensitizer can fluoresce for tumor diagnosis, location and image-guided therapy.^4^ Furthermore, by measuring the fluorescence photobleaching of the photosensitizer, or the detection of ^1^O_2_ phosphorescence emission during the PDT process,^5^ the light dose in the PDT can be accurately administered to prevent under- or over-illumination.

Recently, photosensitizers have been integrated in various nanomaterials including magnetic nanoparticles,^6^ quantum dots,^7^ gold vesicles,^8^ upconversion nanoparticles^9^ and graphene oxide nanosheets^10^ to construct highly efficient theranostic agents. While these agents show encouraging treatment results, there are still three main limitations. The inorganic or metallic nature of most of the nanomaterials has raised great concern about their potential toxicity to normal tissues. To solve this problem, polymer-based materials such as poly(lactic acid) and poly(ethylene oxide) with good biocompatibility are desired because they are typically nontoxic and degraded naturally into safe materials in the body over time.^11^ Another problem is the always-on property of photosensitizers which leads to a high background fluorescence and therapeutic side effects. An efficient way to overcome this problem is to design the photosensitizer with activatable functionality.^12^ The photosensitizer should be switched off in noncancerous tissues but can be switched on by specific cancer-associated stimuli to effectively produce fluorescence and ^1^O_2_. The third problem is that most currently available photosensitizers absorb visible light with limited penetration. Developing a novel photosensitizer with a strong absorbance in the near-infrared (NIR, 700–1000 nm) region can effectively solve this problem,^13^ which improves the homogeneity of the photosensitizer. These limitations and challenges have driven the further design of novel multifunctional photosensitizers and theranostic agents.

**Fig. 1:**
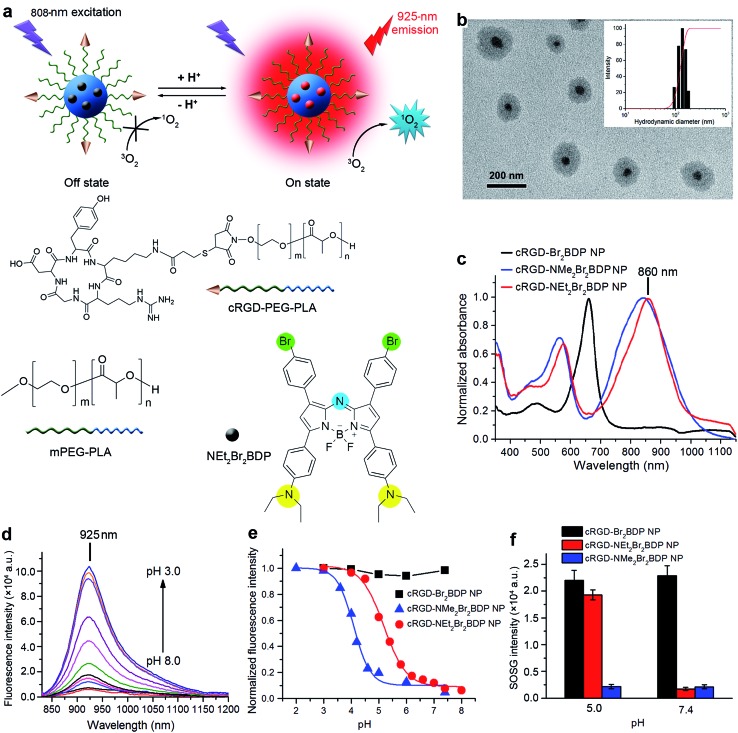
Structure, characterization and optical properties of cRGD-NEt_2_Br_2_BDP NP. (a) Structures and pH-activatable generation of fluorescence and ^1^O_2_ by cRGD-NEt_2_Br_2_BDP NP. (b) TEM image of cRGD-NEt_2_Br_2_BDP NP negatively stained with 2.0% sodium phosphotungstate. Inset: size distribution of cRGD-NEt_2_Br_2_BDP NP determined with DLS. (c) Normalized UV-VIS-NIR absorption spectra of cRGD-Br_2_BDP NP, cRGD-NMe_2_Br_2_BDP NP and cRGD-NEt_2_Br_2_BDP NP. (d) NIR fluorescence spectra of cRGD-NEt_2_Br_2_BDP NP at pH 8.0, 7.4, 7.0, 6.6, 6.2, 5.8, 5.4, 5.0, 4.5, 4.0 and 3.0. (e) pH titration curves of fluorescence intensity of cRGD-Br_2_BDP NP at 685 nm, cRGD-NMe_2_Br_2_BDP NP at 910 nm and cRGD-NEt_2_Br_2_BDP NP at 925 nm. (f) ^1^O_2_ generation of cRGD-Br_2_BDP NP, cRGD-NMe_2_Br_2_BDP NP and cRGD-NEt_2_Br_2_BDP NP at pH 5.0 and 7.4 determined by SOSG fluorescence intensity at 525 nm.

Our previous work reported a nanomicelle as a theranostic agent for simultaneous cancer imaging, PDT and therapeutic monitoring.^14^ However, the fluorescence excitation wavelength was less than 700 nm for monitoring therapeutic efficacy and the photosensitizer was always-on for ^1^O_2_ generation limiting its application. In this regard, we attempt to rationally design a pH-activatable, NIR photosensitizer for the preparation of theranostic agents. Herein, this goal is realized using a newly designed trifunctional photosensitizer, NEt_2_Br_2_BDP, which features an aza-boron-dipyrromethene (aza-BODIPY) structure substituted with diethylaminophenyl and bromophenyl (Fig. 1a). For *in vivo* application, NEt_2_Br_2_BDP is encapsulated in a cyclic RGD peptide-poly(ethylene glycol)-*block*-poly(lactic acid) (cRGD–PEG–PLA) and methoxyl poly(ethylene glycol)-*block*-poly(lactic acid) (mPEG–PLA) nanomicelle to form a theranostic nanoprobe (cRGD-NEt_2_Br_2_BDP NP). The nanoprobe is silent at pH 7.4 in a physiological environment. With the help of the cRGD, it can be selectively taken up by α_v_β_3_ integrin-rich tumor cells into the lysosomes. In these, the nanoprobe is activated by the acidic environment at pH 4.5–5.0 to produce fluorescence at 925 nm for tumor discrimination, and ^1^O_2_ for tumor therapy, under 808 nm irradiation. In addition, therapeutic self-monitoring can be achieved synchronously by monitoring the decrease of fluorescence. This work reports an effective theranostic nanoprobe for highly selective *in vivo* tumor imaging, efficient PDT and therapeutic self-monitoring in the NIR region.

## Results and discussion

### Photophysical properties of NEt_2_Br_2_BDP

Owing to its favourable spectroscopic properties such as high molar absorption coefficient, narrow emission band and excellent photostability,^15^ aza-BODIPY was chosen as the matrix of the photosensitizer NEt_2_Br_2_BDP. The diethylaminophenyl was introduced into the structure of aza-BODIPY as a reactive moiety for pH-activatable NIR ^1^O_2_ generation and fluorescence, and bromophenyl was incorporated to increase the ^1^O_2_ generation efficiency upon pH activation (Scheme S1†) by virtue of the heavy atom effect that enhances intersystem crossing of the excited energy.^16^ For comparison, analogues of the bromophenyl-substituted aza-BODIPY with phenyl (Br_2_BDP) and dimethylaminophenyl (NMe_2_Br_2_BDP) were also synthesized (ESI†). The absorption spectrum of NEt_2_Br_2_BDP displayed a strong peak at 850 nm with a molar absorption coefficient *ε* of 8.64 × 10^4^ M^–1^ cm^–1^ and two weak peaks at 575 nm (*ε* = 4.26 × 10^4^ M^–1^ cm^–1^) and 324 nm (*ε* = 2.62 × 10^4^ M^–1^ cm^–1^) (Fig. S1†). The maximum absorptions of Br_2_BDP and NMe_2_Br_2_BDP were at 655 nm (*ε* = 8.22 × 10^4^ M^–1^ cm^–1^) and 822 nm (*ε* = 8.51 × 10^4^ M^–1^ cm^–1^), respectively, indicating that introduction of the aniline moiety significantly promoted the red shift of absorption.

### Characterization and pH-activatable NIR fluorescence of cRGD-NEt_2_Br_2_BDP NP

Compared to direct administration of free NEt_2_Br_2_BDP, its encapsulation in a cRGD functionalized nanomicelle (Fig. 1a) *via* an emulsion/solvent evaporation method^14^ provided distinct advantages, including better tumor accumulation, improved solubility and sustained drug-release kinetics.^17^ The molecular weights (*M*_w_) of the cRGD–PEG–PLA and mPEG–PLA were determined to be 7.1 and 4.6 kDa respectively by comparing the peak areas of PLA and PEG resonance at 5.20 ppm (–C(=O)–C*H*(–CH_3_–)) and 3.65 ppm (–OC*H*_2_C*H*_2_–) in ^1^H NMR spectra (Fig. S2 and S3†), respectively. The encapsulation efficiency (EE) and loading efficiency (LE) was measured to be 84.8% and 3.2%, respectively (Table S1†). The TEM image of cRGD-NEt_2_Br_2_BDP NP showed a well-dispersed spherical morphology with core–shell structure (Fig. 1b). Its average hydrodynamic diameter, measured by dynamic light scattering (DLS), was 138.4 ± 17.3 nm (Fig. 1b), which did not change for at least four weeks (Fig. S4†), indicating the colloidal stability of the nanoprobe. After encapsulation in the nanomicelle, the absorption of NEt_2_Br_2_BDP was maintained well (Fig. 1c), indicating the structural integrity. The 10 nm red shift of the absorption peak of cRGD-NEt_2_Br_2_BDP NP in PBS compared with those of both free NEt_2_Br_2_BDP and cRGD-NEt_2_Br_2_BDP NP in CHCl_3_ (Fig. S5†) was attributed to the entrapment of NEt_2_Br_2_BDP inside the core of the nanomicelle.^18^ The cRGD-NEt_2_Br_2_BDP NP could be excited at the NIR region to produce a NIR fluorescence emission at 925 nm which was highly pH-dependent (Fig. 1d). The fluorescence at 925 nm was almost undetectable at pH 7.4 with a fluorescence quantum yield *Φ*_F_ of less than 0.002 using indocyanine green (ICG) in DMSO (*Φ*_F_ = 0.13) as a standard (see ESI†). The fluorescence quenching was due to the photoinduced electron transfer from the aniline moiety to the excited fluorophore.^19^ With the increase of solution acidity, the fluorescence at 925 nm increased distinctly, which produced a *Φ*_F_ of 0.18 at pH 4.5. The maintained absorption and pH-dependent fluorescence of the probe indicated a porous structure, which ensured the access of protons to NEt_2_Br_2_BDP. The standard fluorescence pH titration gave an apparent p*K*_a_ value of 5.2 (Fig. 1e), which suggested that the cRGD-NEt_2_Br_2_BDP NP was pH activatable and could respond to a physiologically acidic pH range of 4.5–6.9 (ref. 20) with high sensitivity. Upon 635 nm excitation, the cRGD-Br_2_BDP NP fluoresced at 685 nm (Fig. S6†), and was stable to the solution pH (Fig. 1e), indicating that cRGD-Br_2_BDP NP could be used as an always-on control. In contrast to the designed cRGD-NEt_2_Br_2_BDP NP, the pH-dependent fluorescence of cRGD-NMe_2_Br_2_BDP NP occurred at 910 nm (Fig. S7†), and showed a p*K*_a_ value of 4.1 (Fig. 1e), not matching the physiologically acidic pH range. Thus cRGD-NMe_2_Br_2_BDP NP was used as an always-off control.

### pH-activatable generation of ^1^O_2_

The pH controllable release of ^1^O_2_ induced by cRGD-NEt_2_Br_2_BDP NP under 808 nm irradiation was evaluated with singlet oxygen sensor green (SOSG) as a ^1^O_2_ indicator. This indicator can emit strong fluorescence at 525 nm upon reaction with ^1^O_2_. After exposing the mixed cRGD-NEt_2_Br_2_BDP NP and SOSG solution at pH 7.4 to 808 nm irradiation for 300 s, the SOSG fluorescence intensity at 525 nm did not increase, while the fluorescence intensity of SOSG increased greatly at pH 5.0 under 808 nm irradiation (Fig. S8–S10†). The SOSG intensity increased 11 fold from pH 5.0 to 7.4 (Fig. 1f). The singlet oxygen quantum yields (*Φ*_Δ_) of cRGD-NEt_2_Br_2_BDP NP at different pHs could be measured using a 1,3-diphenylisobenzofuran (DPBF) method (Fig. S11†).^19^ The results indicated that the *Φ*_Δ_ increased from 0.05 at pH 7.4 to 0.56 at pH 5.0, which further confirmed the pH controllable release of ^1^O_2_. The always-on cRGD-Br_2_BDP NP or always-off cRGD-NMe_2_Br_2_BDP NP controls showed negligible pH impact on the SOSG fluorescence intensity after irradiation (Fig. 1f). These results demonstrated that cRGD-NEt_2_Br_2_BDP NP was a potential and promising pH-activatable theranostic agent for cancer treatment.

### Sensitivity of cRGD-NEt_2_Br_2_BDP NP-based NIR imaging

**Fig. 2:**
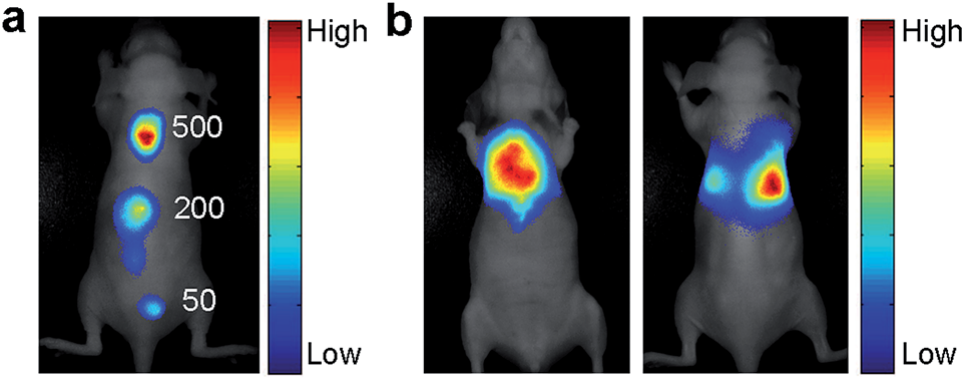
*In vivo* NIR fluorescence imaging sensitivity and depth of U87MG cells labelled with cRGD-NEt_2_Br_2_BDP NP. (a) Imaging sensitivity with reduced cRGD-NEt_2_Br_2_BDP NP-loaded U87MG cell numbers (500, 200 and 50) injected on the back of a nude mouse. (b) Thoracic and dorsal views of a nude mouse with intralumen injection of 1 × 10^4^ cRGD-NEt_2_Br_2_BDP NP-loaded U87MG cells.

NIR fluorescence facilitates bioimaging and detection because it is less susceptible to interference from tissue autofluorescence.^21^ The sensitivity of the cRGD-NEt_2_Br_2_BDP NP-based fluorescence imaging was studied in BALB/c nude mice (Fig. 2a). The α_v_β_3_ integrin-rich human glioblastoma U87MG tumor cells were used as a model and labelled with cRGD-NEt_2_Br_2_BDP NP. After 500, 200 and 50 cRGD-NEt_2_Br_2_BDP NP-labelled U87MG cells were subcutaneously injected into the back of a nude mouse, the mouse was subjected to imaging on a Maestro EX *in vivo* imaging system. The U87MG cells displayed distinct fluorescence at the injection sites while no fluorescence signals were observed in other regions of the mouse body. The detectable cell number down to 50 cells, lower than the detection limit of 98 cells of infrared-emitting long-persistence luminescent nanoparticles-based cell tracking,^22^ was clearly visualized, revealing good sensitivity. The sensitivity was attributed to the use of NIR fluorescence as this is less susceptible interference from tissue autofluorescence. The penetrability of NIR fluorescence was further examined with a mouse treated with an intralumen injection of cRGD-NEt_2_Br_2_BDP NP-labelled U87MG cells (Fig. 2b), which indicated that the NIR fluorescence could penetrate through the thoracic cavity of the live nude mouse and was detectable on its back. The thickness of the mouse body was about 2 cm, and the penetration depth of the pH activatable cRGD-NMe_2_Br_2_BDP NP was at least 1 cm. These results confirmed that the excitation and emission light of the nanoprobe possessed acceptable penetration depth for the imaging of tumor cells.

### *In vivo* imaging on subcutaneous U87MG tumor-bearing mice

**Fig. 3:**
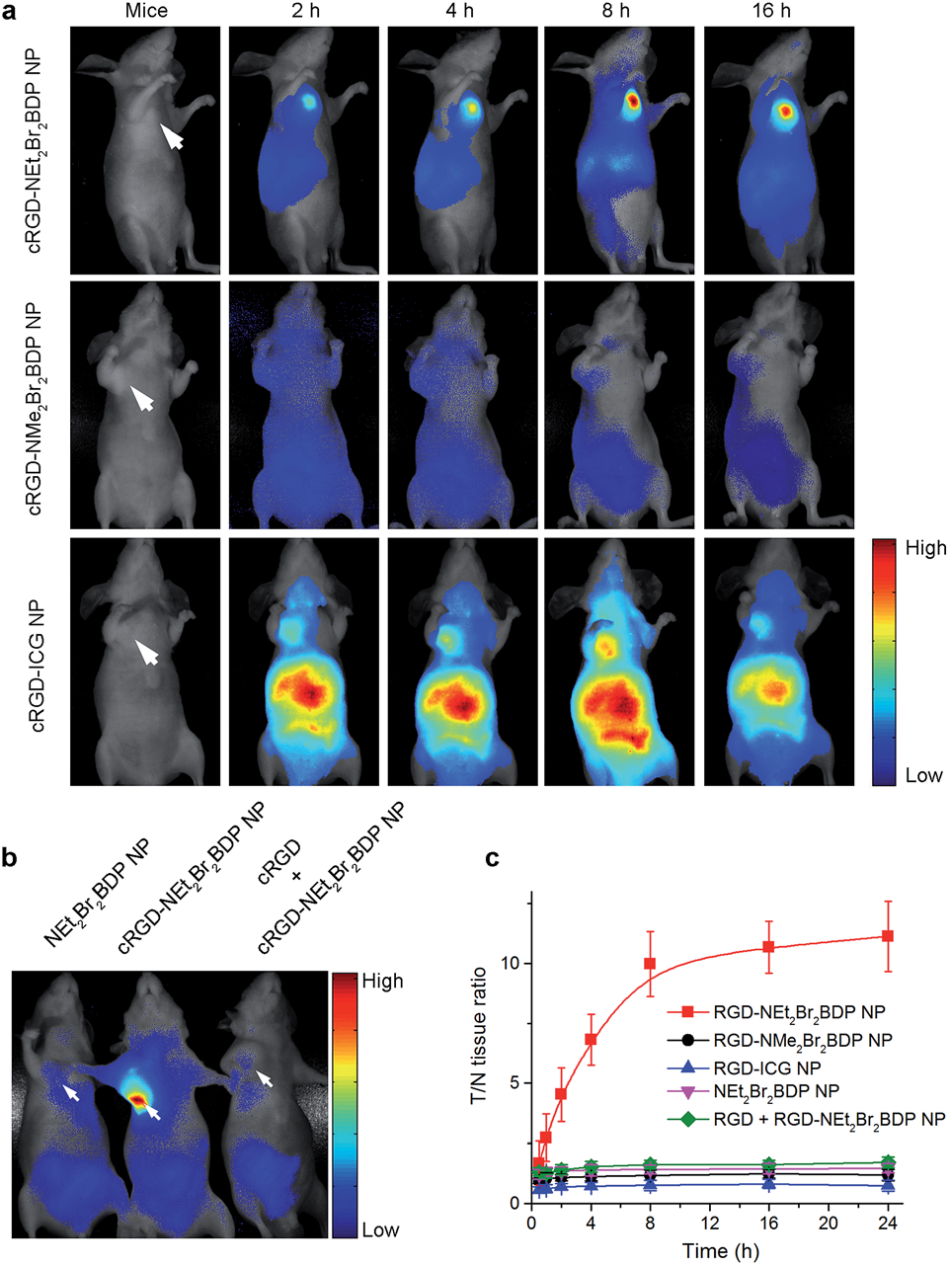
*In vivo* NIR fluorescence tumor imaging. The arrows show the tumor sites. (a) Time-dependent *in vivo* NIR fluorescence images of subcutaneous U87MG tumor-bearing mice after intravenous injection of 20 mg kg^–1^ pH-activatable cRGD-NEt_2_Br_2_BDP NP, always-off cRGD-NMe_2_Br_2_BDP NP as a negative control or always-on cRGD-ICG NP as a positive control. (b) *In vivo* NIR fluorescence image of subcutaneous U87MG tumor-bearing mice at 8 h post-injection of 20 mg kg^–1^ NEt_2_Br_2_BDP NP or cRGD-NMe_2_Br_2_BDP NP, and a competition group. In the competition group, a blocking dose of cRGD peptide (25 mg kg^–1^) is injected 30 min before cRGD-NEt_2_Br_2_BDP NP administration. (c) *In vivo* time-dependent T/N tissue ratios after injection of different nanoprobes. Data are presented as means ± SD (*n* = 6).

The blood circulation curve of cRGD-NEt_2_Br_2_BDP NP in mice showed the biphasic clearance profile with a rapid clearance during the first 5 h followed by a continuous and slow elimination rate (Fig. S12†). The blood circulation half-life was found to be 4.8 h, indicating a long persistence of cRGD-NEt_2_Br_2_BDP NP in the bloodstream to corroborate its stability *in vivo*. The *in vivo* tumor imaging was investigated in mice bearing a subcutaneous U87MG tumor xenograft. The U87MG tumor-bearing mice were intravenously injected with cRGD-NEt_2_Br_2_BDP NP, cRGD-NMe_2_Br_2_BDP NP or cRGD-ICG NP, and subjected to *in vivo* imaging at different time points to explore the function of pH activation (Fig. 3a).

To explore the targeting effect of cRGD, the U87MG tumor-bearing mice were also injected intravenously with NEt_2_Br_2_BDP NP without cRGD functionalization and subjected to imaging (Fig. 3b). As an additional control, a blocking dose of cRGD was injected before the cRGD-NEt_2_Br_2_BDP NP administration in one mouse (Fig. 3b). Two hours after injection, the tumor tissue in the cRGD-NEt_2_Br_2_BDP NP-treated mouse could be distinguished from the surrounding normal tissues (Fig. 3a). The ratio of fluorescence at tumor to normal tissue (T/N tissue ratio) increased gradually and reached a maximum (10 fold) at 8 h postinjection, and a high fluorescence contrast could be maintained even 16 h after postinjection (Fig. 3c), demonstrating the targeted delivery, pH-activatable fluorescence and long retention of cRGD-NEt_2_Br_2_BDP NP in tumor tissue. *Ex vivo* imaging clearly showed that the specific activation of cRGD-NEt_2_Br_2_BDP was at the tumor tissue over other organs including heart, liver, spleen, lung, kidney, stomach, intestine and muscle (Fig. S13†). Conversely, the always-off cRGD-NMe_2_Br_2_BDP NP treated mouse, which was used as a negative control, showed negligible fluorescence in the tumor (Fig. 3a) and other organs (Fig. S14†). This could be explained by the p*K*_a_ of 4.1 of this photosensitizer resulting in the inactivation of cRGD-NMe_2_Br_2_BDP NP. For the always-on cRGD-ICG NP treated mouse, used as a positive control, although the tumor displayed fluorescence, the interferences from normal tissues were serious (Fig. 3a), and the high fluorescence background was also observed in lung, liver and kidneys (Fig. S15†), leading to a low T/N tissue ratio (less than 0.8) at all time points (Fig. 3c). Compared to the always-off cRGD-NMe_2_Br_2_BDP NP and always-on cRGD-ICG NP, the 10 fold T/N tissue ratio verified the advantages of the pH-activatable function of cRGD-NEt_2_Br_2_BDP NP by reducing the signal background from the normal tissues. For the NEt_2_Br_2_BDP NP and free cRGD competition controls, no fluorescence was observed in tumor (Fig. 3b), which confirmed the contribution of cRGD in tumor targeting for pH activation.

### *In vivo* PDT evaluation

**Fig. 4:**
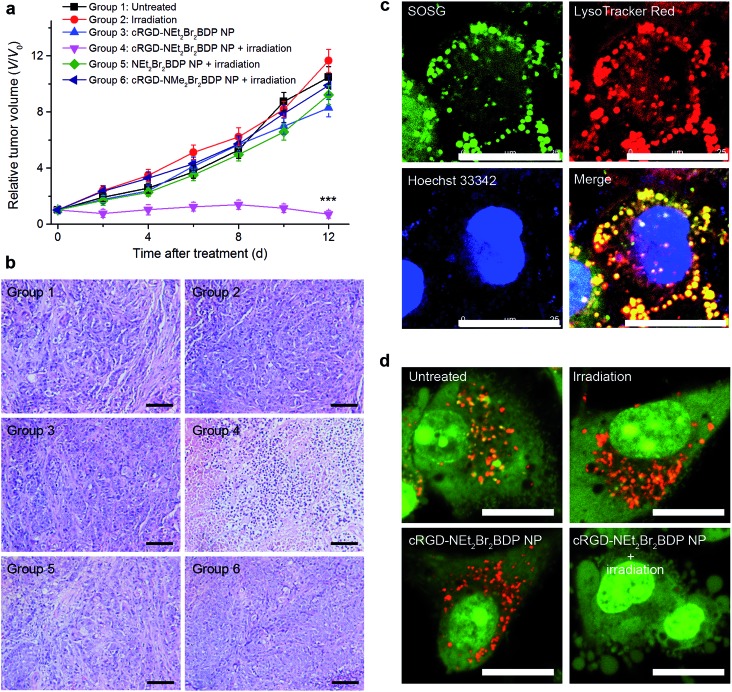
*In vivo* and *in vitro* NIR PDT against tumors. (a) Tumor growth curves of different treatment groups. Data are presented as means ± SD (*n* = 6, ***P < 0.001 compared to other groups using one-way ANOVA). (b) H&E stained images of tumor tissue sections from different groups at 8 days after treatment. Scale bars: 100 μm. (c) Subcellular localization of ^1^O_2_ generated during cRGD-NEt_2_Br_2_BDP NP-mediated PDT with SOSG, LysoTracker Red and Hoechst 33342 staining. Scale bars: 25 μm. (d) Lysosomal stability observed with confocal fluorescence images of AO-stained U87MG cells after different treatments. Scale bars: 20 μm.

For *in vivo* PDT treatment, the U87MG tumor-bearing mice were randomly divided into 6 groups of the untreated mice (group 1), treated with irradiation (group 2), cRGD-NEt_2_Br_2_BDP NP injected (group 3), cRGD-NEt_2_Br_2_BDP NP injected and then treated with irradiation (group 4), NEt_2_Br_2_BDP NP injected and then irradiated (group 5), or cRGD-NMe_2_Br_2_BDP NP injected and then irradiated (group 6). 8 h after injection, the corresponding treatment was conducted. The irradiation was performed with a NIR 808 nm laser at an irradiance of 100 mW cm^–2^ for 300 s. The therapeutic effects after treatment were assessed by monitoring the change of relative tumor volumes (Fig. 4a) and hematoxylin and eosin (H&E) staining (Fig. 4b). No tumor growth inhibition or tumor tissue necrosis was observed in the group of mice in the absence of irradiation and/or cRGD-NEt_2_Br_2_BDP NP, which indicated that irradiation with a low irradiance of 100 mW cm^–2^ had little photothermal effect and the cRGD-NEt_2_Br_2_BDP NP had little dark toxicity. In marked contrast, the tumor growth for group 4 was remarkably suppressed, and the tumor tissue showed obvious necrosis, indicating that cRGD-NEt_2_Br_2_BDP NP could be effectively activated under NIR irradiation to produce a strong phototoxicity to the tumor. Group 5 did not show a therapeutic effect in tumor tissue due to the lack of cRGD targeting for pH activation, and group 6 showed rapid tumor growth because the cRGD-NMe_2_Br_2_BDP NP could not be activated by physiologically acidic pH under irradiation. These results confirmed the high therapeutic effect of cRGD-NEt_2_Br_2_BDP NP-mediated PDT.

The potential toxicity or side effects are always a great concern for theranostic agents used in medicine. At the cellular level, the NEt_2_Br_2_BDP before and after irradiation showed negligible toxicity (Fig. S16†). The mice treated with the NP-mediated PDT did not show apparent body weight loss (Fig. S17†). At 12 days after treatment, the PDT-treated mice and the age-matched healthy mice without treatment were sacrificed, and the major organs including heart, liver, spleen, lung, and kidney were collected for H&E staining to evaluate toxic effect. No noticeable sign of organ damage was observed on the H&E-stained organ slices (Fig. S18†), which suggested the safety of cRGD-NEt_2_Br_2_BDP NP for *in vivo* PDT application.

### Therapeutic mechanism of cRGD-NEt_2_Br_2_BDP NP-mediated PDT

In order to understand the therapeutic mechanism down to the subcellular level, the cRGD-NEt_2_Br_2_BDP NP-loaded U87MG cells were incubated with SOSG to study the subcellular localization of ^1^O_2_. After 808 nm irradiation, strong SOSG fluorescence was observed which overlapped with the LysoTracker Red (Fig. 4c), suggesting that ^1^O_2_ generation occurred in the lysosomes. Previous work had demonstrated that ^1^O_2_ production in the lysosome induced lysosomal membrane permeabilization and subsequent release of cathepsins into cytosol to trigger cell-death pathways.^14,19^ To further confirm the lysosomal destruction, acridine orange (AO) was employed as an integrity indicator of lysosomes, which emits red fluorescence in lysosomes and green fluorescence in the cytosol and nuclei.^23^ The lysosomes in the U87MG cells displayed bright red fluorescence in the absence of cRGD-NEt_2_Br_2_BDP NP and/or irradiation (Fig. 4d), suggesting that the lysosomal compartments were intact. After the cRGD-NEt_2_Br_2_BDP NP-loaded cells were irradiated, the red AO fluorescence disappeared (Fig. 4d), which indicated the destruction of the lysosomal membrane. After the U87MG cells were treated with the always-off cRGD-NMe_2_Br_2_BDP NP, the irradiation with a 808 nm laser did not damage the lysosomes (Fig. S19a†), which excluded the photothermal effect during irradiation. Moreover, the addition of vitamin C as a ^1^O_2_ scavenger could efficiently prevent the damage to the lysosomes (Fig. S19b†). Therefore, the generated ^1^O_2_ from the cRGD-NEt_2_Br_2_BDP NP-mediated PDT induced the cell death in a lysosome-associated pathway.

### Therapeutic self-monitoring

**Fig. 5:**
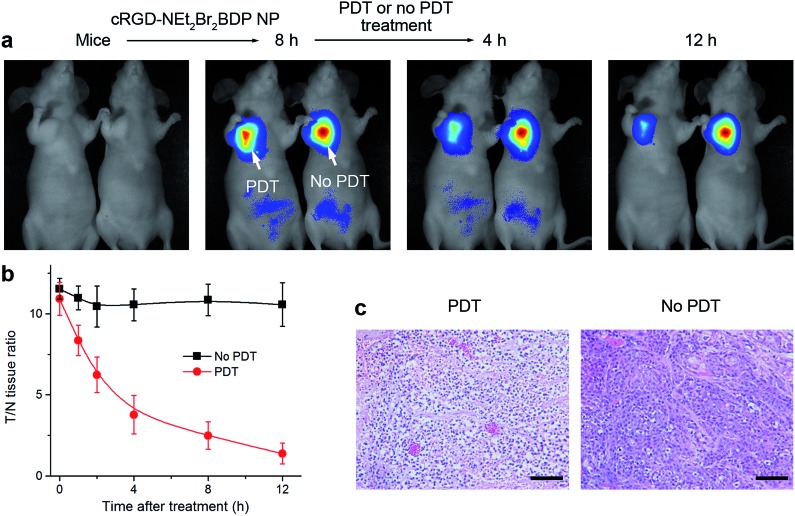
*In situ* and real-time self-monitoring of PDT efficacy. (a) *In vivo* PDT and therapeutic monitoring on subcutaneous U87MG tumor-bearing mice with cRGD-NEt_2_Br_2_BDP NP. 8 h post-injection, the 808 nm irradiation is implemented to the tumor of the left mouse, and the tumor on the right mouse is kept in dark as a control. (b) Time-dependent T/N tissue ratios of subcutaneous U87MG tumor-bearing mice with or without PDT. Data are presented as means ± SD (*n* = 6). (c) H&E stained images of the U87MG tumor tissue from the mice treated with or without PDT. Scale bars: 100 μm.

Owing to the intrinsic property of the pH-activatable fluorescence of cRGD-NEt_2_Br_2_BDP NP, the function of therapeutic self-monitoring during the PDT process could be realized by fluorescence imaging (Fig. 5a). Two groups of mice bearing U87MG tumors were intravenously injected with the nanoprobe. 8 h after the injection, they displayed a T/N tissue ratio of more than 10 fold (Fig. 5b). With the pH-activatable fluorescence guiding, the group of mice was irradiated with an 808 nm laser at 100 mW cm^–2^ for 300 s, and another group of mice was kept in the dark as a control. After irradiation, the therapeutic self-monitoring was performed by observing the fluorescence change of cRGD-NEt_2_Br_2_BDP NP. The fluorescence intensity from the irradiated tumor decreased gradually (Fig. 5a) and almost disappeared with a 1.4 fold T/N tissue ratio after 12 h (Fig. 5b). In contrast, the changes in both fluorescence (Fig. 5a) and T/N tissue ratio (Fig. 5b) for the unirradiated tumor were negligible over the same period. In order to investigate the physiological transformation along with the fluorescence change after PDT, H&E staining of the two tumors was performed. Prominent necrosis was observed in the PDT-treated tumor tissue, while necrosis was indiscernible in the unirradiated tumor (Fig. 5c). Therefore, the cRGD-NEt_2_Br_2_BDP NP displayed not only highly selective tumor imaging and effective therapy functions, but also a self-monitoring capability for the evaluation of therapeutic efficacy.

## Experimental section

### Preparation and characterization of cRGD-NEt_2_Br_2_BDP NP

An emulsion/solvent evaporation method was used for the preparation of cRGD-NEt_2_Br_2_BDP NP.^14^ In brief, 21 mg of cRGD-PEG–PLA and mPEG–PLA copolymers with a mixing ratio of 5% (w/w%) and 0.8 mg of NEt_2_Br_2_BDP were dissolved in 2 mL of CH_2_Cl_2_, which was then added to 4 mL of 2% Pluronic® F-127 aqueous solution. The mixture was sonicated using a probe sonicator (Scientz Biotechnology Co. Ltd, China) for 30 s at 180 W output. The formed emulsion was added dropwise to 20 mL of a magnetically stirred 0.5% Pluronic® F-127 aqueous solution. After stirring for 1 h, the remaining organic solvent was removed in a rotary evaporator at reduced pressure at 30 °C. The cRGD-NEt_2_Br_2_BDP NP solution was filtrated through a Millipore syringe filter (pore size 0.45 μm) to remove unencapsulated NEt_2_Br_2_BDP aggregates and further purified by centrifugation at 21 000×*g* for 15 min using a centrifugal filter with cutoff *M*_w_ of 100 kDa (Millipore, MA). After purification the cRGD-NEt_2_Br_2_BDP NP solution was freeze-dried. Similar procedures were used to prepare the cRGD-Br_2_BDP NP, cRGD-NMe_2_Br_2_BDP NP, cRGD-ICG NP and NEt_2_Br_2_BDP NP, in which the nanocarrier was composed of mPEG–PLA. The EE and LE of the photosensitizer loaded in the cRGD functionalized nanomicelles were determined by UV-VIS-NIR absorption spectroscopy. The EE and LE were expressed according to the following formulas: EE (%) = ((weight of loaded photosensitizer)/(weight of initially added photosensitizer)) × 100; LE (%) = ((weight of loaded photosensitizer)/(total weight of nanomicelle)) × 100. DLS and TEM measurements were performed to examine the size distribution and morphology of cRGD-NEt_2_Br_2_BDP NP.

### *In vivo* tumor imaging

All animal operations were in accord with institutional animal use and care regulations approved by the Model Animal Research Center of Nanjing University (MARC). Specific pathogen-free (SPF) BALB/c nude mice, 5–6 weeks of age, were purchased from Shanghai Laboratory Animal Center, Chinese Academy of Sciences (SLACCAS) and bred in an axenic environment. Glioblastoma tumor models were established in BALB/c nude mice by injecting 1 × 10^6^ U87MG cells subcutaneously into the selected positions. Tumors were then allowed to grow to 8–10 mm in diameter. For the blood circulation time assay of cRGD-NEt_2_Br_2_BDP NP, the mice were fasted but had free access to water for 16 h, and were then intravenously injected with 20 mg kg^–1^ cRGD-NEt_2_Br_2_BDP NP *via* tail vein. After injection for 0.5, 1, 3, 6, 12, 24 and 48 h, these mice were sacrificed, respectively, and the blood samples were obtained. With a standard curve method, the blood circulation time of the cRGD-NEt_2_Br_2_BDP NP was determined by measuring the fluorescence of cRGD-NEt_2_Br_2_BDP NP under acidic condition. To demonstrate the contribution of pH activation, 20 mg kg^–1^ pH-activatable cRGD-NEt_2_Br_2_BDP NP, always-off cRGD-NMe_2_Br_2_BDP NP or always-on cRGD-ICG NP was intravenously injected into the U87MG tumor-bearing mice (*n* = 6) *via* a tail vein. *In vivo* tumor imaging was then performed on a Maestro EX *in vivo* imaging system with an excitation of 735 nm and collected emission range of 800–950 nm at 2, 4, 8 and 16 h postinjection. At 24 h postinjection, the mice were euthanized to obtain the organs and tumor tissues for imaging. To elucidate the role of α_v_β_3_-mediated endocytosis, a group of mice (*n* = 6) were intravenously injected with 20 mg kg^–1^ NEt_2_Br_2_BDP NP at 8 h and another group of mice (*n* = 6) were intravenously injected with cRGDyk (25 mg kg^–1^) 30 min before cRGD-NEt_2_Br_2_BDP NP administration. During the injection and image acquisition process, the mice were anesthetized with 2.5% isoflurane in oxygen delivered at a flow rate of 1.5 L min^–1^. T/N tissue ratios were determined by comparing the average fluorescence intensities in the tumor and the whole body except the tumor site using Maestro 3.0 software.

### *In vivo* PDT

*In vivo* cRGD-NEt_2_Br_2_BDP NP-mediated PDT was performed using U87MG tumor-bearing mice. The mice were randomly divided into 6 groups for the following treatments and each group contained six mice: group 1, untreated; group 2, irradiated with an 808 nm laser for 300 s at an irradiance of 100 mW cm^–2^ (irradiation); group 3, intravenously injected with 20 mg kg^–1^ cRGD-NEt_2_Br_2_BDP NP (cRGD-NEt_2_Br_2_BDP NP); group 4, intravenously injected with 20 mg kg^–1^ cRGD-NEt_2_Br_2_BDP NP and irradiated with an 808 nm laser at an irradiance of 100 mW cm^–2^ for 300 s after 8 h (cRGD-NEt_2_Br_2_BDP NP + irradiation); group 5, intravenously injected with 20 mg kg^–1^ NEt_2_Br_2_BDP NP and irradiated with an 808 nm laser at an irradiance of 100 mW cm^–2^ for 300 s after 8 h (NEt_2_Br_2_BDP NP + irradiation); group 6, intravenously injected with 20 mg kg^–1^ cRGD-NMe_2_Br_2_BDP NP and irradiated with an 808 nm laser at an irradiance of 100 mW cm^–2^ for 300 s after 8 h (cRGD-NMe_2_Br_2_BDP NP + irradiation). The mice from different treatment groups were monitored by measuring the tumor volume every two days for 12 days after treatment. The greatest longitudinal diameter (length) and the greatest transverse diameter (width) of each tumor were determined using a vernier caliper, and the tumor volume was calculated using length × width^2^ × 0.5.^24^

### *In vivo* therapeutic self-monitoring

For self-monitoring of therapeutic efficacy, two groups of mice bearing U87MG tumors were intravenously injected with 20 mg kg^–1^ cRGD-NEt_2_Br_2_BDP NP *via* a tail vein. 8 h postinjection, one group of mice (*n* = 6) was irradiated with an 808 nm laser at an irradiance of 100 mW cm^–2^ for 300 s and another group of mice (*n* = 6) was kept in the dark as a control. The therapeutic monitoring was performed *in vivo* by observing the fluorescence change after irradiation by a Maestro EX *in vivo* imaging system. The mice were sacrificed 24 h after irradiation to examine the histopathology of the tumors by H&E staining under a BX51 optical microscope (Olympus, Japan) in a blind fashion by a pathologist.

## Conclusions

This work reports the successful synthesis of an effective theranostic nanoprobe named cRGD-NEt_2_Br_2_BDP NP to achieve highly selective *in vivo* tumor imaging, efficient PDT and therapeutic self-monitoring in the NIR region by encapsulating a newly designed trifunctional photosensitizer NEt_2_Br_2_BDP in the cRGD-functionalized nanomicelle. The cRGD-NEt_2_Br_2_BDP NP with little dark toxicity but strong phototoxicity can target to tumor cells *via* cRGD and be activated by physiologically acidic pH to produce fluorescence and ^1^O_2_ for theranostics. The fascinating advantage of the nanoprobe is the NIR implementation beyond 800 nm, which significantly improves the imaging sensitivity and increases penetration depth for PDT *via* a lysosomal cell-death pathway. By monitoring the fluorescence decrease in tumor region after PDT, the therapeutic efficacy is reflected *in situ* and in real time, which provides a valuable and convenient self-feedback function for PDT efficacy tracking. Therefore, this rationally designed and carefully engineered nanoprobe offers a new paradigm for precise tumor theranostics and may provide novel opportunities for future clinical cancer treatment.
