# Supplementary material for: A pH-activatable and aniline-substituted photosensitizer for near-infrared cancer theranostics
Source: Chem Sci. 2015 Jul 13;6(10):5969–77. doi: 10.1039/c5sc01721a (PMC5529996; doi:10.1039/c5sc01721a)
Supplement: Supplementary file 1 [file SC-006-C5SC01721A-s001.pdf]

This journal is © The Royal Society of Chemistry 2015

Electronic Supplementary Information for

A pH-activatable and aniline-substituted photosensitizer for near-infrared cancer theranostics

Jiangwei Tian, Jinfeng Zhou, Zhen Shen, Lin Ding, Jun-Sheng Yu and Huangxian Ju*

State Key Laboratory of Analytical Chemistry for Life Science, State Key Laboratory of Coordination Chemistry, School of Chemistry and Chemical Engineering, Nanjing University, Nanjing 210093, P.R. China. Fax/Tel.: +86 25 83593593; E-mail: hxju@nju.edu.cn

Contents

1. Materials and Reagents……………………………………………………2

2. Apparatus……………………………………………………………………2

3. Statistical Analysis…………………………………………………………3

4. Supplemental Methods………………………………………………………3

4.1 Synthesis and Characterization of NMe_2Br_2BDP and NEt_2Br_2BDP…………………3

4.2 Fluorometric Analysis………………………………………………7

4.3 Singlet Oxygen ($^1O_2$) Detection…………………………………7

4.4 Cell Culture……………………………………………………………8

4.5 Intracellular $^1O_2$ Colocalization Assay…………………………8

4.6 Lysosomal Stability Assay……………………………………………8

4.7 MTT Assay……………………………………………………………8

4.8 *In Vivo* Toxicity Assay………………………………………………9

5. Supplemental Table…………………………………………………………9

6. Supplemental Figures………………………………………………………10

7. Supplemental References…………………………………………………16

1. Materials and Reagents

Unless otherwise noted, all reagents were purchased from commercial suppliers and directly used without further purification. 4-Bromobenzaldehyde, 1,3-diphenylisobenzofuran (DPBF), rose bengal (RB) and trifluorobonetherate (BF_3Et_2O) were purchased from Alfa Aesar (Ward Hill, MA, USA). Nitromethanol was purchased from Beijing InnoChem Science & Technology Co. Ltd. 1-(4-(Diethylamino)phenyl)ethanone and 1-(4-(dimethylamine)phenyl)ethanone were purchased from TCI Chemical Industry Co. Ltd (Shanghai, China). Dichloromethane was distilled from sodium and were stored in a Teflon-capped tube under nitrogen gas prior to use. Indocyanine green (ICG), 3-[4,5-dimethylthiazol-2-yl]-2,5-diphenyltetrazolium bromide (MTT), vitamin C and methoxy-polyethylene glycol (mPEG-OH, Mw= 2.0 KDa) were purchased from Sigma-Aldrich (St. Louis, MO, USA). Maleimide-polyethylene glycol (Mal-PEG-OH, Mw= 2.9 KDa) was purchased from Shanghai Yare Biotech, Inc (Shanghai, China). Singlet oxygen sensor green (SOSG, S-36002), LysoTracker® Red, Hoechst 33342 and acridine orange (AO) were obtained from Invitrogen (Carlsbad, CA, USA). Annexin V-FITC/propidium iodide (PI) cell apoptosis kit was obtained from KeyGen Biotech. Co. Ltd. (Nanjing, China). Cyclic RGD peptide c(RGDyK) (Mw= 619.6) was synthesized by ChinaPeptides Co., Ltd. (Shanghai, China). Methoxyl poly(ethylene glycol)-*block*-poly(lactic acid) (mPEG-PLA), c(RGDyK) conjugated poly(ethylene glycol)-*block*-poly(lactic acid) (cRGD-PEG-PLA)[S1] and BF_2chelate of 3-(4-bromophenyl)-*N*-(3-(4-bromophenyl)-5-phenyl-1*H*-pyrrol-2-yl)-5-phenyl-2*H*-pyrrol-2-imine (Br_2BDP)[S2] were synthesized as described previously. Ultrapure water was prepared using a Millipore simplicity system (Millipore, Bedford, USA).

2. Apparatus

^{1}H NMR spectra were recorded with a Bruker 500 MHz spectrometer.Chemical shifts (δ) were referenced with $CDCl_3$ (δ = 7.26 ppm) as the internal standard. Chemical shifts were reported as part per million (ppm) in (δ) scale downfield from tetramethylsilane (TMS). Coupling constants (J) were reported in Hertz (Hz). The mass spectra were recorded on Finnigan MAT TSQ 7000 for ESI-MS. MALDI-TOF MS data were measured on Bruker Daltonics autoflex^{II}. UV-VIS-NIR spectra were recorded on a SHIMADZU UV-3600 spectrophotometer. Steady-state fluorescence spectra were measured on an FLS-920 spectrofluorometer (Edinburgh Instruments Ltd., UK). The morphology of cRGD-NEt_2Br_2BDP NP was performed with transmission electron microscopy (TEM, JEM-200CX, JEOL, Japan) operating at an accelerated voltage of 200 kV. The sample for TEM measurement was prepared by dropping the solution onto a carbon-coated copper grid following negative staining with 2.0% (w/v) phosphotungstic acid. The particle size and size distribution were measured by dynamic light scattering (DLS) using a Mastersizer 2000 particle size analyzer (Malvern Instruments, U.K.). Confocal fluorescence imaging experiments were performed on a confocal laser scanning microscope (CLSM; TCS SP5, Leica, Germany). *In vivo* fluorescence imaging experiments were performed on a Maestro EX in-vivo imaging system (CRI, Inc.). The irradiation was performed with an 808-nm NIR laser (LWIR808nm, Laserwave Ltd, China) at an irradiance of 100 mW cm^{-2}. Light was focused on the tumor to give a final beam of 10 mm in diameter and the energy density homogenization of irradiaion spot was greater than 90%.

3. Statistical Analysis

Data were expressed as means ± SD from at least three experiments. Statistical analysis was carried out using a statistics program (GraphPad Prism; GraphPad Software). One-way ANOVA was used to compare the treatment effects. $P < 0.05$ was considered to be statistically significant.

4. Supplemental Methods

4.1 Synthesis and Characterization of NMe_2Br_2BDP and NEt_2Br_2BDP

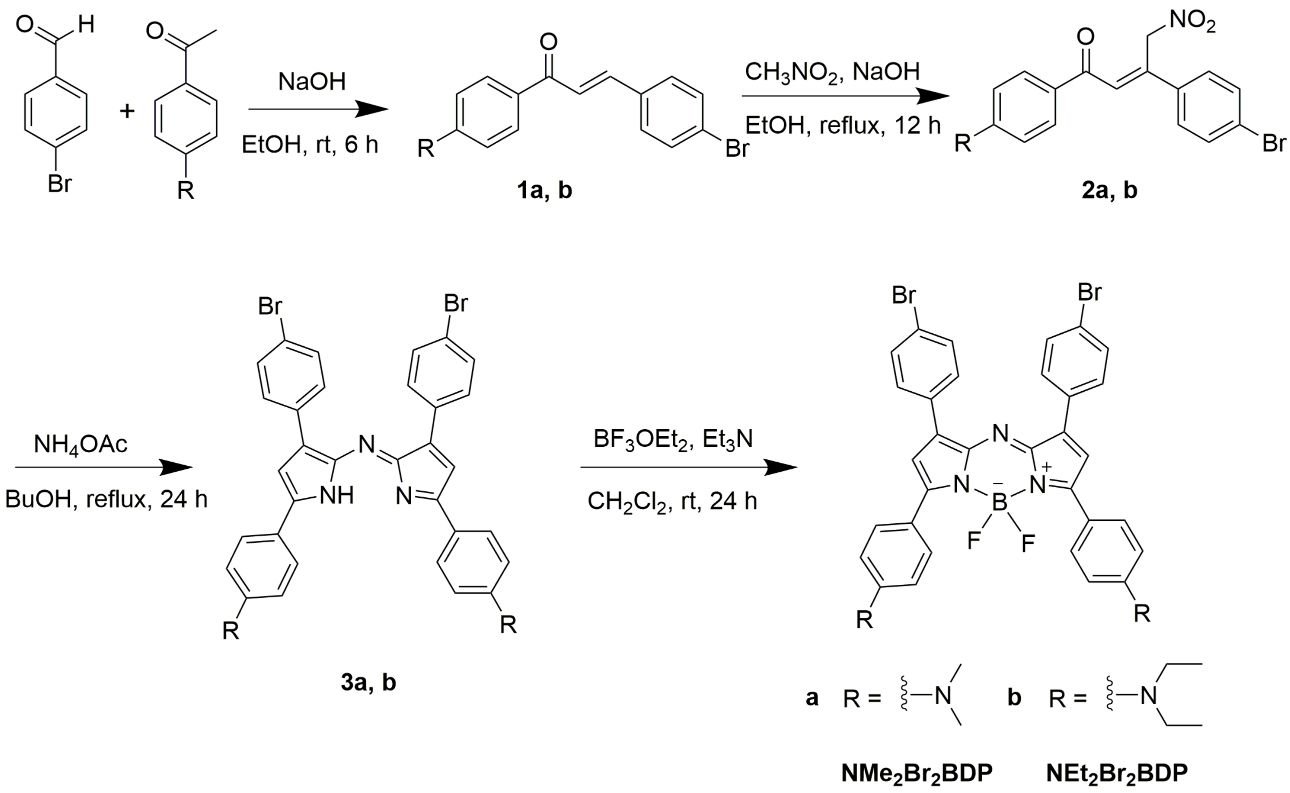

Scheme S1. Synthesis of NMe_2Br_2BDP and NEt_2Br_2BDP

3-(4-Bromophenyl)-1-(4-(dimethylamino)phenyl)prop-2-en-1-one(1a). 4-Bromobenzaldehyde (1.85 g, 10 mmol) and 1-(4-(dimethylamine)phenyl)ethanone (1.63g, 10 mmol) were dissolved in ethanol (10 mL), 10% NaOH (10 mL) was slowly dropped in the above solution, the reaction mixture was stirred at room temperature until a heavy precipitate was formed (6 h). The precipitate was collected on a filter and washed with cold MeOH to afford the yellow compound (2.97 g, 90%). ^{1}H NMR (500 MHz, $CDCl_3$, δ): 8.00 (d, J = 10 Hz, 2H, Ar H), 7.69 (d, J = 15 Hz, 2H, Ar H), 7.48-7.58 (m, 5H, Ar H), 6.74 (d, J = 10 Hz, 2H, CH), 3.09 (s, 6H, CH_3). ESI-MS (m/z): $[M+H]^+$ calcd for $C_{17}H_{17}BrNO$ 332.05; found, 332.50.

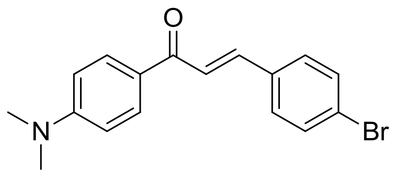

3-(4-Bromophenyl)-1-(4-(diethylamino)phenyl)prop-2-en-1-one (1b). 4-Bromobenzaldehyde (1.85 g, 10 mmol) and 1-(4-(diethylamino)phenyl)ethanone (1.91 g, 10 mmol) were dissolved in ethanol (10 mL), 10% NaOH (10 mL) was slowly dropped in the above solution and stirred at room temperature until a heavy precipitate was formed (6 h). The precipitate was collected on a filter and washed with cold MeOH to afford the yellow compound (2.76 g, 76%). ^{1}H NMR (500 MHz, $CDCl_3$, δ): 7.98 (d, J = 7.5Hz, 2H, Ar H), 7.70 (d, J = 13Hz, 2H, Ar H), 7.48-7.58 (m, 5H, Ar H), 6.68 (d, J = 5.5Hz, 2H, CH), 3.44 (q, J = 6 Hz, 4H, CH_2), 1.22 (t, J = 6 Hz,6H, CH_3). ESI-MS (*m/z*): $[M+H]^+$ calcd for $C_{19}H_{21}BrNO$ 382.06; found, 382.58.

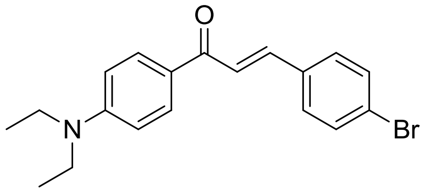

3-(4-Bromophenyl)-1-(4-(dimethylamino)phenyl)-4-nitrobutan-1-one (2a). A solution of chalcone (1.32 g, 4 mmol), nitromethanol (4.88 g, 80 mmol) and NaOH (32 mg, 0.8 mmol) in ethanol (3 mL) was heated at 60 ^oC for 12 h. After cooling to room temperature, the solvent was removed in vacuo and the oily residue obtained was dissolved in ethyl acetate and washed with water. The combined organic layer was washed with brine, dried over sodium sulfate, and concentrated to give the target compound as a yellow oily residue. This product was used in the next without further purification (1.68 g, 67%).^{1}H NMR (500 MHz, $CDCl_3$, δ): 7.82 (d, J = 9.0 Hz, 2H, Ar H), 7.44 (d, J = 8.5 Hz, 2H, Ar H), 7.18 (d, J = 8.0 Hz, 2H, Ar H), 6.62 (d, J = 9.5 Hz, 2H, Ar H), 4.81-4.85 (m, 1H, CH), 4.62-4.66 (m, 1H, CH), 4.11-4.21 (m, 1H, CH), 3.24-3.35 (m, 2H, CH_2), 3.09 (s, 6H, CH_3). ESI-MS (*m/z*): $[M+H]^+$ calcd for $C_{18}H_{20}BrN_2O_3$ 393.06; found, 393.42.

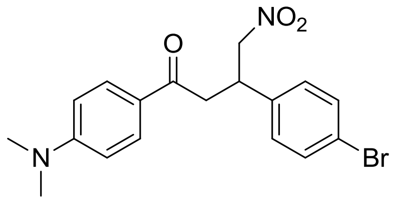

3-(4-Bromophenyl)-1-(4-(diethylamino)phenyl)-4-nitrobutan-1-one (2b). A solution of chalcone (1.44 g, 4 mmol), nitromethanol (4.88 g, 80 mmol) and NaOH (32 mg, 0.8 mmol) in ethanol (3 mL) was heated at 60 ^oC for 12 h. After cooling to room temperature, the solvent was removed and the oily residue obtained was dissolved in ethyl acetate and washed with water. The combined organic layer was washed with brine, dried over sodium sulfate, and concentrated to give the target compound as a yellow oily residue (1.68 g, 72%). This product was used in the next without further purification.^{1}H NMR (500 MHz, $CDCl_3$, δ): 7.79 (d, J = 9.0 Hz, 2H, Ar H), 7.44 (d, J = 8.5 Hz, 2H, Ar H), 7.17 (d, J = 8.5 Hz, 2H, Ar H), 6.58 (d, J =9.0Hz, 2H, Ar H), 4.81-4.85 (m, 1H, CH), 4.62-4.66 (m, 1H, CH), 4.10-4.21 (m, 1H, CH), 3.42 (q, J = 7.0 Hz, 4H, CH_2), 1.20 (t, J = 6 Hz,6H, CH_3). ESI-MS (*m/z*): $[M+H]^+$ calcd for $C_{20}H_{23}BrN_2NaO_3$, 443.08; found, 443.58.

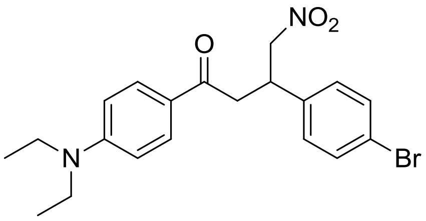

3-(4-Bromophenyl)-*N*-(3-(4-bromophenyl)-5-(4-(dimethylamino)phenyl)-2*H*-pyrrol-2-ylidene)-5-(4-(dimethylamino)phenyl)-1*H*-pyrrol-2-amine (3a). A 50 mL round-bottomed flask was charged with **2a** (1.17 g, 3 mmol), ammonium acetate (8.08 g, 105 mmol), and butanol (30 mL) and heated under reflux for 24 h. During the course of the reaction, the product was precipitated from the reaction mixture. The reaction was cooled to room temperature and filtered and isolated solid washed with ethanol to yield the product as a blue-black solid (509 mg, 49%).^{1}H NMR (500 MHz, $CDCl_3$, δ): 8.42 (d, *J* = 8.0 Hz, 2H, Ar H), 7.93 (d, *J* = 7.0Hz, 2H, Ar H), 7.83 (d, *J* = 7.5 Hz, 2H, Ar H),7.52 (d, *J* =8.0 Hz, 2H, Ar H), 7.45 (d, *J* = 7.5 Hz, 2H, Ar H), 7.37 (d, *J* = 8.0Hz, 2H, Ar H), 7.15 (s, 2H, CH), 6.83 (d, *J* = 7.5Hz, 4H, Ar H), 3.12 (s, 12H, CH_3). MALDI-TOF (*m/z*): calcd for $C_{36}H_{31}Br_2N_5$, 693.09; found, 693.53.

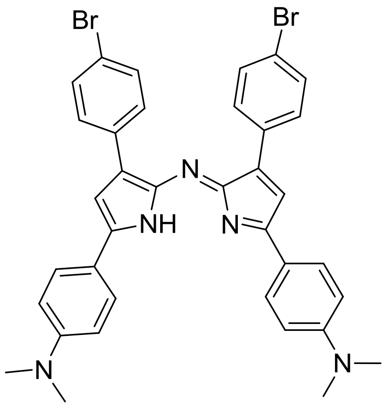

3-(4-Bromophenyl)-*N*-(3-(4-bromophenyl)-5-(4-(diethylamino)phenyl)-2*H*-pyrrol-2-ylidene)-5-(4-(diethylamino)phenyl)-1*H*-pyrrol-2-amine (3b). A 50 mL round-bottomed flask was charged with **2b** (1.26 g, 3 mmol), ammonium acetate (8.08 g, 105 mmol), and butanol (30 mL) and heated under reflux for 24 h. After cooling to room temperature, the formed precipitate was filtered, washed with cold ethanol and diethyl ether to give a blue-solid product (483 mg, 43%).^{1}H NMR (500 MHz, $CDCl_3$, δ): 7.92 (d, *J* = 8.5 Hz, 4H, Ar H), 7.79 (d, *J* = 8.5 Hz, 4H, Ar H), 7.52 (d, *J* = 8.0 Hz, 4H, Ar H), 7.07 (s, 2H, CH), 6.78 (d, *J* = 8.5 Hz, 4H, Ar H), 3.48 (q, *J* = 7.0 Hz, 8H, CH_2), 1.26 (t, *J* = 7.0 Hz, 12H, CH_3). MALDI-TOF (*m/z*): calcd for $C_{40}H_{39}Br_2N_5$, 749.15; found, 749.71.

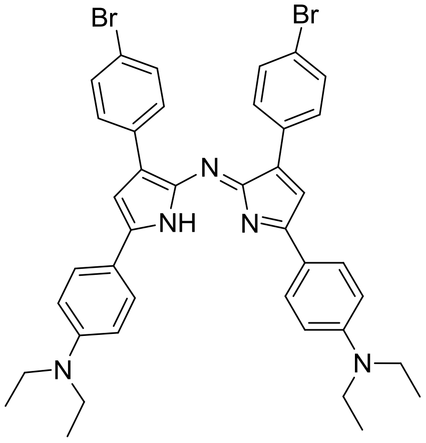

BF$_2$chelate of 3-(4-bromophenyl)-*N*-(3-(4-bromophenyl)-5-(4-(dimethylamino)phenyl)-2H-pyrrol-2-ylidene)-5-(4-(dimethylamino)phenyl)-1*H*-pyrrol-2-amine (NMe$_2$Br$_2$BDP). Compound **3a** (104 mg, 0.15 mmol) was dissolved in dry CH_2Cl_2 (10 mL) in the dark under Ar, and dissopropylethylamine (0.55 mL, 3 mmol) was then added. After the solution was stirred for 10 min, BF_3Et_2O (0.37 mL, 3 mmol) was added, which was stirred at room temperature for 24 h. The reaction mixture was extracted with chloroform and washed with water and brine. The organic layer was dried with anhydrous Na_2SO_4. The solvent was removed under reduced pressure, and the residue was purified by column chromatography on silica gel eluting with CH_2Cl_2/hexane (1/1) to give the product NMe$_2$Br$_2$BDP as purple solid (97 mg, 87%). ^{1}H NMR (500 MHz, $CDCl_3$, δ): 8.15 (d, *J* = 8.0 Hz, 4H, Ar H), 7.91 (d, *J* = 8.5 Hz, 4H, Ar H), 7.57 (d, *J* = 8.5 Hz, 4H, Ar H), 6.98-7.08 (m, 6H, CH, Ar H), 6.98 (m, 4H, Ar H), 3.14 (s, 12H, CH_3).MALDI-TOF (*m/z*): calcd for $C_{36}H_{30}BBr_2F_2N_5$, 741.09; found, 740.44.

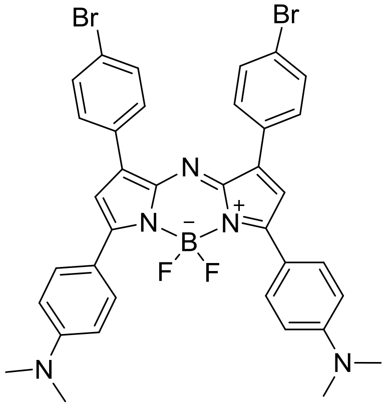

BF$_2$chelate of 3-(4-bromophenyl)-*N*-(3-(4-bromophenyl)-5-(4-(diethylamino)phenyl)-2H-pyrrol-2-ylidene)-5-(4-(diethylamino)phenyl)-1*H*-pyrrol-2-amine (NEt$_2$Br$_2$BDP). Compound **3b** (112 mg, 0.15 mmol) was dissolved in dry CH_2Cl_2 (10 mL) in the dark under Ar, and dissopropylethylamine (0.55 mL, 3 mmol) was then added. After the solution was stirred for 10 min, BF_3Et_2O (0.37 mL, 3 mmol) was added, which was stirred at room temperature for 24 h. The reaction mixture was extracted with chloroform and washed with water and brine. The organic layer was dried with anhydrous Na_2SO_4. The solvent was removed under reduced pressure, and the residue was purified by column chromatography on silica gel eluting with CH_2Cl_2/hexane (1/1) to give the product NEt$_2$Br$_2$BDPas purple solid (91 mg, 76%).^{1}H NMR (500 MHz, $CDCl_3$, δ): 8.10 (d, *J* = 8.0 Hz, 4H, Ar H), 7.91 (d,

J = 10.5 Hz, 4H, Ar H), 7.56 (d, J = 10.5 Hz, 4H, Ar H), 6.63-6.84 (m, 6H, CH, Ar H), 3.24-3.71 (m, 4H, CH_2), 1.25 (t, J = 7.5Hz, 12H, CH_3). MALDI-TOF (*m/z*): calcd for $C_{40}H_{38}BBr_2F_2N_5$, 797.15; found, 797.07.

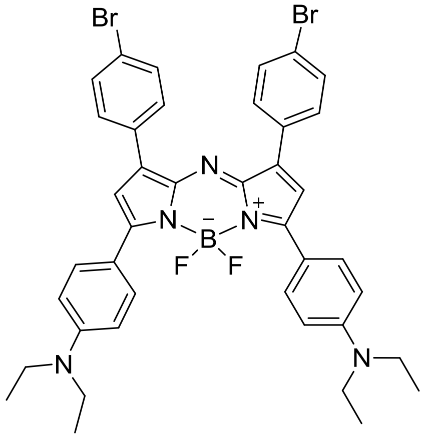

4.2 Fluorometric Analysis. Fluorescence spectra of the nanoprobes (0.25 mg mL^{-1}) at different pHs were recorded on an FLS-920 spectrofluorometer equipped with a red-sensitive Hamamatsu R5509-73 photomultiplier and a C9940-02 cooler. The slit width was 5.0 nm for excitation and emission. The fluorescence quantum yields (Φ_F) of cRGD-NEt_2Br_2BDP NP at different pHs were measured using ICG in DMSO (Φ_F = 0.13) as the standard and calculated with the following equation,[S3]

$$\boldsymbol{\Phi}_{\mathbf{F(x)}} = \boldsymbol{\Phi}_{\mathbf{F(std)}} \left(\frac{\boldsymbol{A}_{\mathbf{std}}}{\boldsymbol{A}_{\boldsymbol{x}}}\right)\left(\frac{\boldsymbol{I}_{\mathbf{x}}}{\boldsymbol{I}_{\mathbf{std}}}\right)\left(\frac{\boldsymbol{\eta}_{\boldsymbol{x}}}{\boldsymbol{\eta}_{\mathbf{std}}}\right)^{2}$$

where subscript x designates cRGD-NEt_2Br_2BDP NP, subscript std designates ICG, A stands for the absorbance at excitation wavelength, I stands for the integrated fluorescence intensity, and η stands for the refractive index of the solvent in the measurements.

4.3 Singlet Oxygen ($^1O_2$) Detection. The nanoprobesof 0.25 mg mL^{-1} were inbucated with 1.0 μM SOSG as a $^1O_2$ fluorescent probe at pH 5.0 or 7.4. Afterwards, the solution was irradiated with a 808-nm laser at an irradiance of 100 mW cm^{-2} for 300 s. The fluorescence intensity of SOSG was measured by a spectrofluorometer with excitation at 480 nm and emission at 525 nm. The slit width was 3.0 nm for excitation and emission. Singlet oxygen quantum yields (Φ_Δ) of cRGD-NEt_2Br_2BDP NP at different pHs were detected through monitoring the oxidation of DPBF.[S4,S5] Briefly an oxygen-saturated solution of cRGD-NEt_2Br_2BDP NP containing 30 μM DPBF was prepared in the dark and irradiated with a 561-nm laser at a power of 100 mW cm^{-2} in an interval of 30 s. DPBF oxidation was monitored by UV-Vis spectrophotometer. The Φ_Δ values were calculated with a relative method using RB in ethanol (Φ_Δ = 0.79)[S6] as the standard and the following equation,[S4]

$$\Phi_{\Delta(x)} = \Phi_{\Delta(std)}\left(\frac{S_x}{S_{std}}\right)\left(\frac{F_{std}}{F_x}\right)$$

where the subscripts x and std designate the cRGD-NEt_2Br_2BDP NP and RB, respectively, S stands for the slope of plot of the absorbance of DPBF (at 418 nm) vs irradiation time. F stands for the absorption correction factor, which is given by $F = 1 - 10^{-A}$ (A represents the absorbance of cRGD-NEt_2Br_2BDP NP and RB at 561 nm).

4.4 Cell Culture. Human glioblastoma U87MG cell line was purchased from American Type Culture Collection (ATCC, Manassas, VA). The cells were cultured in Dulbecco's Modified Eagle Medium (DMEM) supplemented with 10% fetal bovine serum (FBS) and 1% penicillin/streptomycin in a humidified atmosphere of 5% CO_2 at 37 ℃.

4.5 Intracellular $^1O_2$ Colocalization Assay. U87MG cells were seeded at a density of 1 × 10^4 cells $well^{-1}$ in 35-mm CLSM-special cell culture dishes and incubated for 24 h at 37 ℃ under 5% CO_2. The cRGD-NEt_2Br_2BDP NP was dispersed into DMEM cell-culture media with a concentration of 0.25 mg mL^{-1}, and then added into the culture dish. After incubation for 4 h, the cells were washed three times with PBS to remove the nonuptake nanoprobe. For colocalization imaging assay of cRGD-NEt_2Br_2BDP NP-mediated generation of $^1O_2$, the cells were stained with 1.0 μM SOSG, LysoTracker® Red and Hoechst 33342 for 10 min, respectively, and irradiated with a 808 nm laser for 300 s at an irradiance of 100 mW cm^{-2}. Then confocal fluorescence imaging experiments were performed on CLSM. SOSG was excited at 488 nm with an argon ion laser and the emission was collected from 500 to 550 nm. LysoTracker® Red was excited with a 532 nm diode laser and the emission was collected from 560 to 650 nm. Hoechst 33342 was excited with a violet 405 nm diode laser and the emission was collected from 420 to 480 nm. A 60 × oil immersion objective lens was used and the fluorescence images were analyzed by Leica Application Suite Advanced Fluorescence (LAS-AF) software.

4.6 Lysosomal Stability Assay. For lysosomal stability assay during cRGD-NEt_2Br_2BDP NP-mediated PDT, U87MG Cells were seeded at a density of 1 × 10^4 cells $well^{-1}$ in 35-mm CLSM-special cell culture dishes, incubated for 24 h at 37 ℃ under 5% CO_2, and randomly divided into six groups for the following treatments: group 1, untreated; group 2, irradiated with 808 nm laser for 300 s at an irradiance of 100 mW cm^{-2} (irradiation); group 3, incubated with 0.25 mg mL^{-1} cRGD-NEt_2Br_2BDP NP for 4 h (cRGD-NEt_2Br_2BDP NP); group 4, incubated with 0.25 mg mL^{-1} cRGD-NEt_2Br_2BDP NP for 4 h and irradiated with 808 nm laser for 300 s at an irradiance of 100 mW cm^{-2} (cRGD-NEt_2Br_2BDP NP + irradiation); group 5, incubated with 0.25 mg mL^{-1} cRGD-NMe_2Br_2BDP NP for 4 h and irradiated with 808 nm laser for 300 s at an irradiance of 100 mW cm^{-2} (cRGD-NMe_2Br_2BDP NP + irradiation); group 6, incubated with 0.25 mg mL^{-1} cRGD-NEt_2Br_2BDP NP and 2.5 mM vitamin C for 4 h, and irradiated with 808 nm laser for 300 s at an irradiance of 100 mW cm^{-2} (cRGD-NEt_2Br_2BDP NP +vitamin C + irradiation). After treatment, the cells were stained with 5.0 μM AO for 15 min and performed on CLSM. The images were collected from 515−545 nm (green) and 610−640 nm (red) at an excitation wavelength of 488 nm.

4.7 MTT assay. U87MG cells were seeded into 96-well cell-culture plate at 1 × 10^4 cells per well, followed by incubation at 37 °C for 24 h. After rinsing with PBS, 100 μL NEt_2Br_2BDP before and after irradiation at concentrations of 0.05, 0.1, 0.25, 0.5, 1.0, 2.5, 5.0, 10 and 20 μM was added to the wells, respectively. The cells were incubated for 48 h at 37 ℃ under 5% CO_2. Then, 20 μL of 5 mg mL^{-1} MTT solution in PBS was added to each well. After incubating the cells for 4 h, the medium containing unreacted dye was removed carefully, and 200 μL DMSO was added to each well to dissolve blue formazan. After 1 h the absorbance was measured with a Bio-Rad microplate readerat a wavelength of 490 nm. The cell viability was then determined by the following equation:

cell viability (%)= (mean of absorbance value of treatment group)/(mean of absorbance value of control) × 100.

4.8 *In Vivo* Toxicity Assay. The U87MG tumor-bearing mice were intravenously injected with 20 mg kg^{-1} cRGD-NEt_2Br_2BDP NP and irradiated with 808 nm laser to perform PDT. 12 days after treatment, the PDT-treated mice and the age-matched healthy mice without treatment were sacrificed by CO_2 asphyxiation for necropsy. Major organs including heart, liver, spleen, lung and kidney were harvested to examine the histopathology of organs by H&E staining.

4. Supplemental Table

Table S1. Encapsulation efficiency (EE) and loading efficiency (LE) of Br_2BDP, NMe_2Br_2BDP or NEt_2Br_2BDP in the cRGD functionalized nanomicelle.

	EE (%)	LE (%)
Br_2BDP	80.2 ± 2.3	3.0 ± 0.3
NMe_2Br_2BDP	81.6 ± 2.7	3.1 ± 0.3
NEt_2Br_2BDP	84.8 ± 2.4	3.2 ± 0.2

5. Supplemental Figures

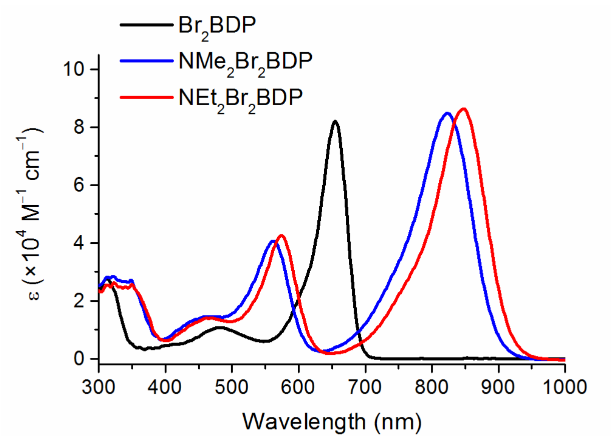

Figure S1. UV-VIS-NIR absorption spectra of Br_2BDP, NMe_2Br_2BDP and NEt_2Br_2BDP in $CHCl_3$.

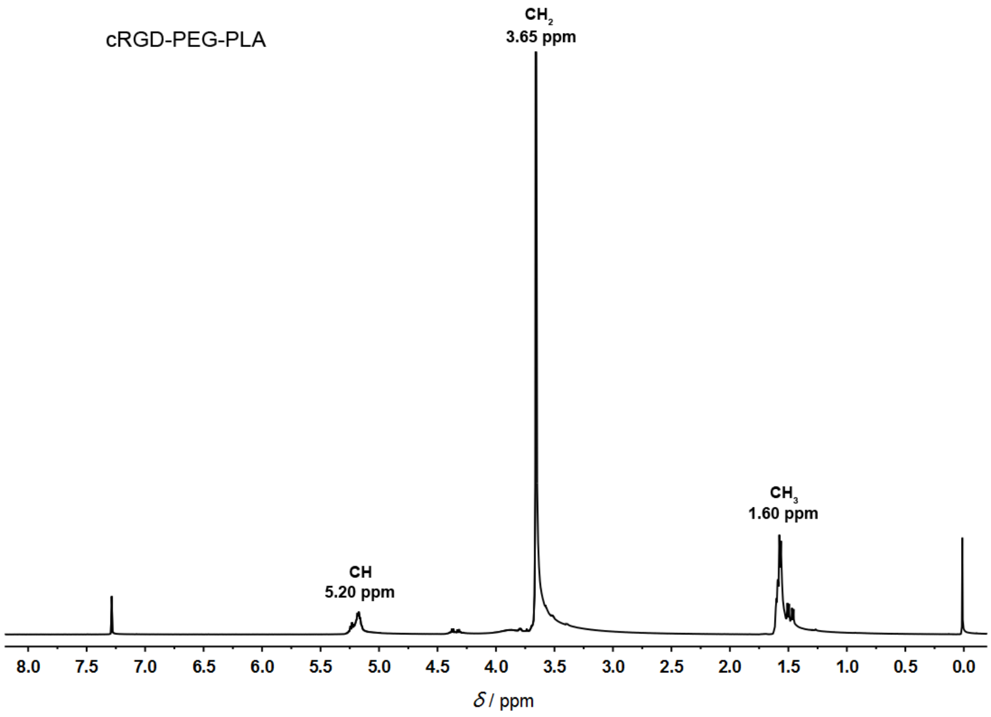

Figure S2. ^{1}H NMR spectrum of cRGD-PEG-PLA in $CDCl_3$.

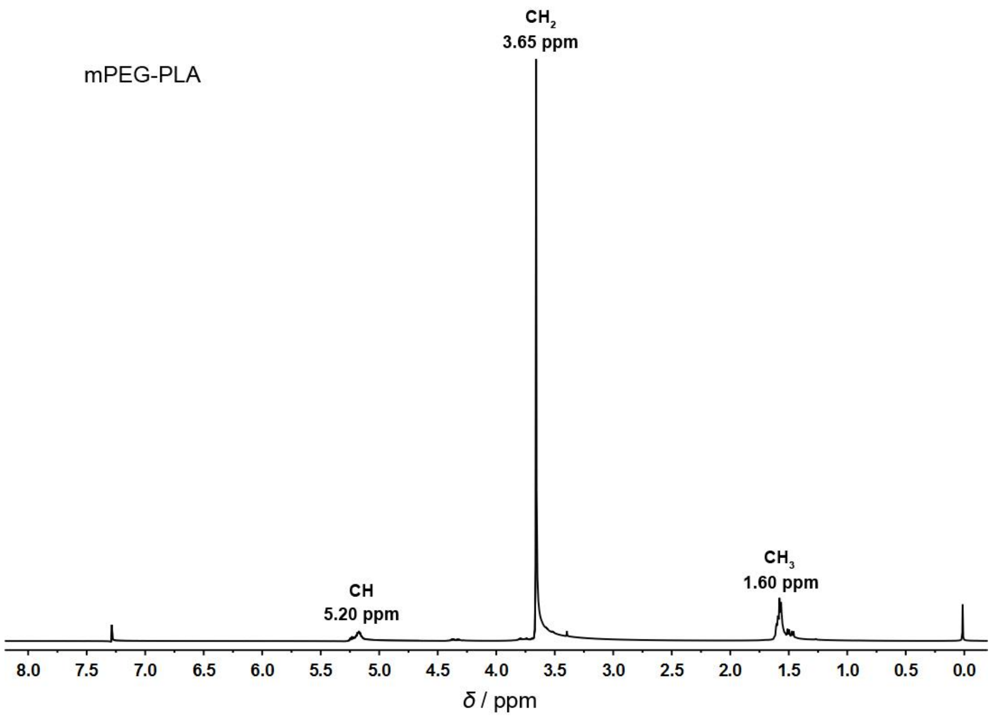

Figure S3. ^{1}H NMR spectrum of mPEG-PLA in $CDCl_3$.

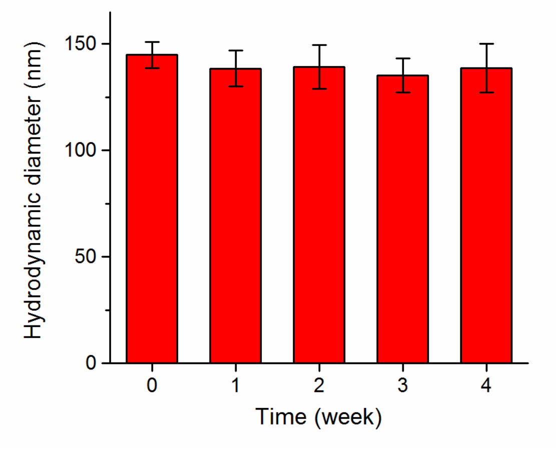

Figure S4. Colloid stability test of cRGD-NEt_2Br_2BDP NP determined by dynamic light scattering.

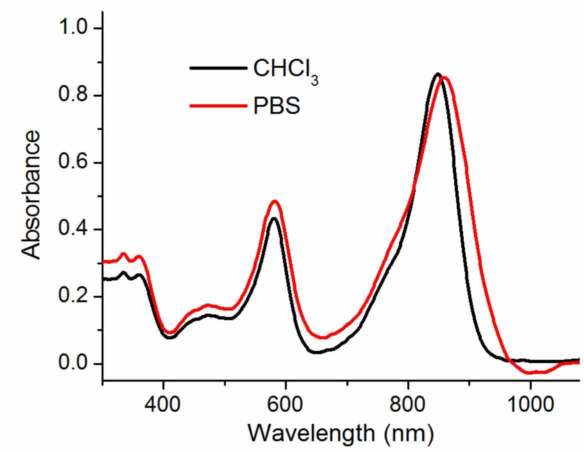

Figure S5. UV-VIS-NIR absorption spectra of 0.25 mg mL^{-1} cRGD-NEt_2Br_2BDP NP in $CHCl_3$ and PBS.

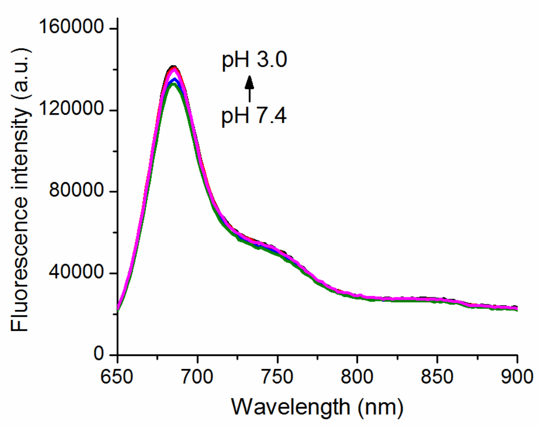

Figure S6. Fluorescence emission spectra of cRGD-Br_2BDP NP at pH 7.4, 6.0, 5.0, 4.0 and 3.0.

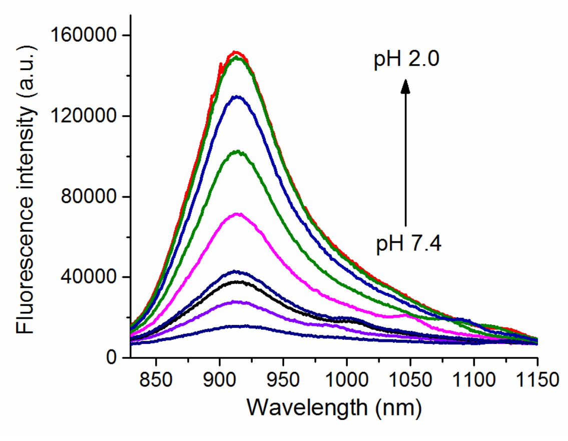

Figure S7. Fluorescence emission spectra of cRGD-NMe_2Br_2BDP NP at pH 7.4, 6.0, 5.0, 4.6, 4.2, 4.0, 3.6, 3.0 and 2.0.

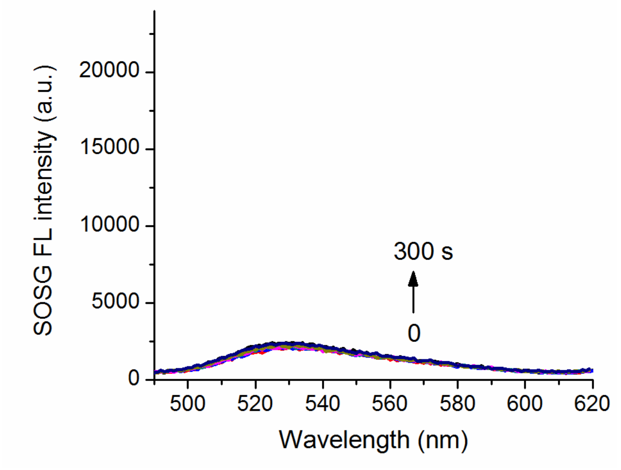

Figure S8. Time-course SOSG fluorescence (FL) emission spectra of the cRGD-NEt_2Br_2BDP NP solution at pH 7.4 under 808 nm irradiation.

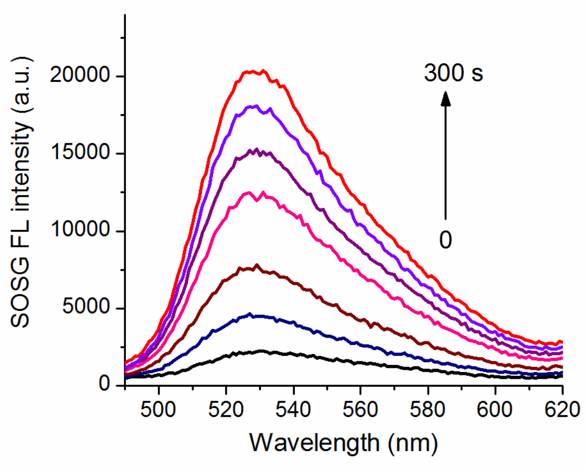

Figure S9. Time-course SOSG FL emission spectra of cRGD-NEt_2Br_2BDP NP at pH 5.0 under 808 nm irradiation.

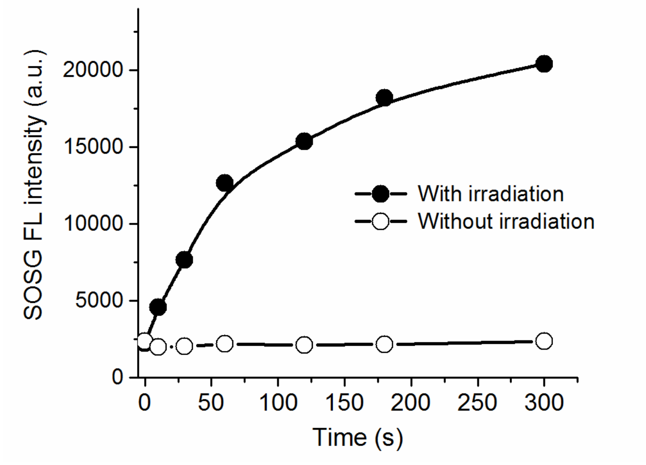

Figure S10. Time-course 525 nm FL intensity of SOSG to determine $^1O_2$ generated by cRGD-NEt_2Br_2BDP NP at pH 5.0 with or without 808 nm irradiation.

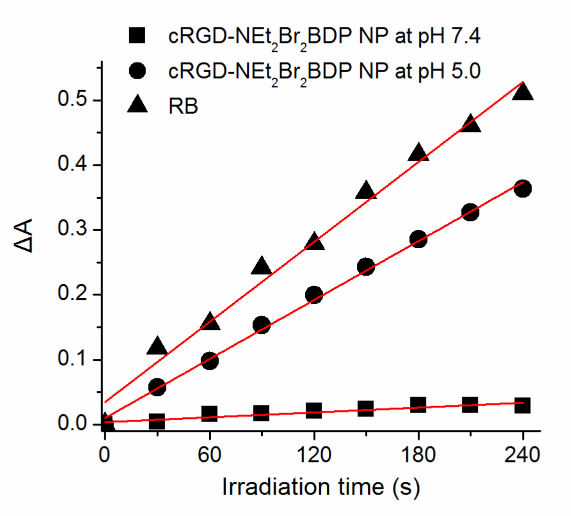

Figure S11. Plots of change in absorbance of DPBF at 418 nm vs irradiation time in the presence of cRGD-NEt_2Br_2BDP NP at pH 7.4 and 5.0 against RB in ethanol as the standard.

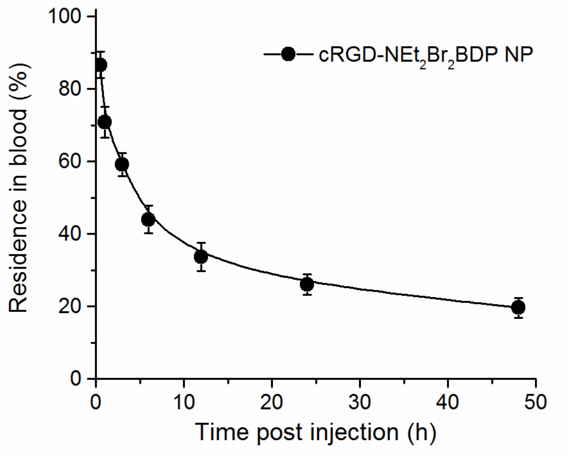

Figure S12. Blood circulation curve of cRGD-NEt_2Br_2BDP NP (*n* = 3).

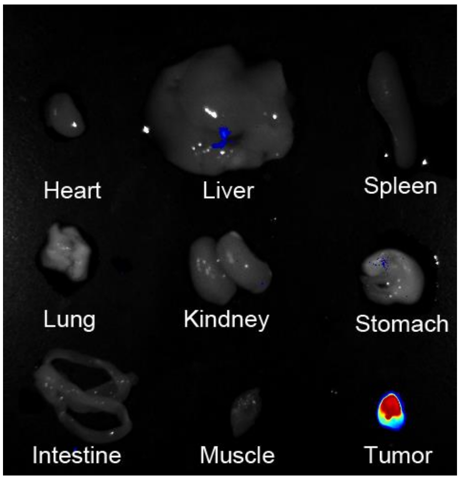

Figure S13. Fluorescence image of major organs and tumor after 24-h postinjection of cRGD-NEt_2Et_2BDP NP.

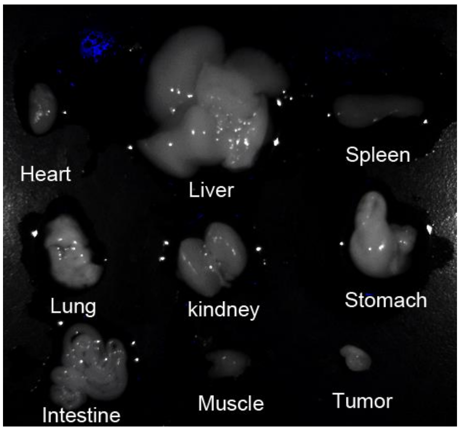

Figure S14. Fluorescence image of major organs and tumor after 24-h postinjection of cRGD-NMe_2Br_2BDP NP.

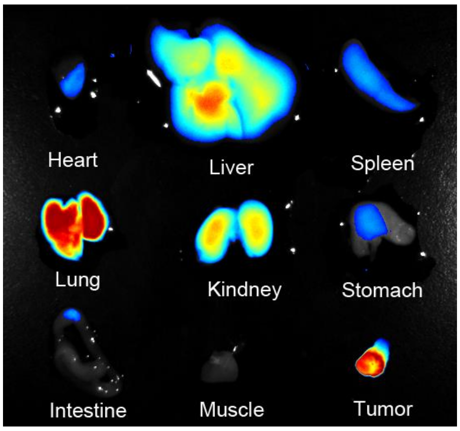

Figure S15. Fluorescence image of major organs and tumor after 24-h postinjection of cRGD-ICG NP.

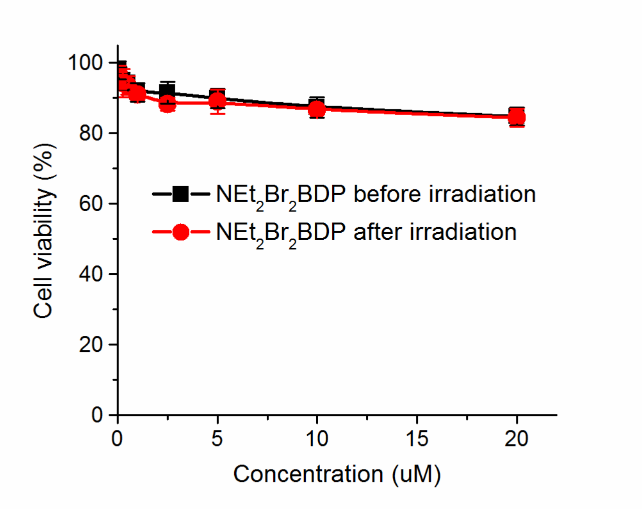

Figure S16. MTT assay for U87MG cells after treatment with NEt_2Br_2BDP or irradiated NEt_2Br_2BDP at different concentrations.

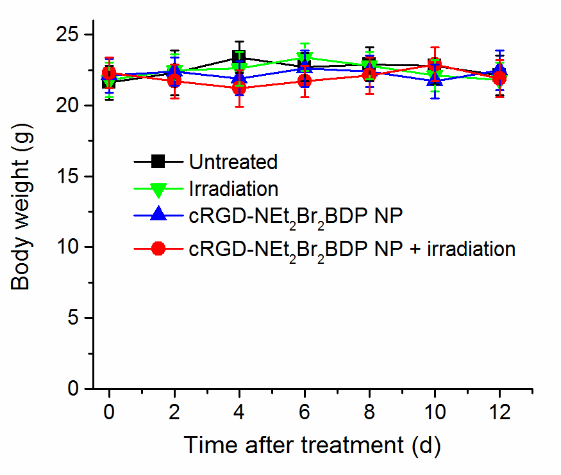

Figure S17. Body weight changes of U87MG tumor-bearing mice after various treatments (*n* = 6).

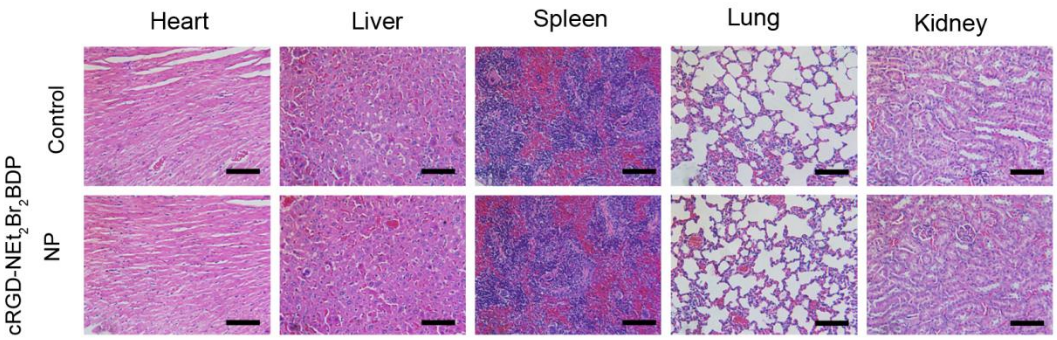

Figure S18. H&E stained images of major organs for *in vivo* toxicity assay. Scale bars: 100 μm.

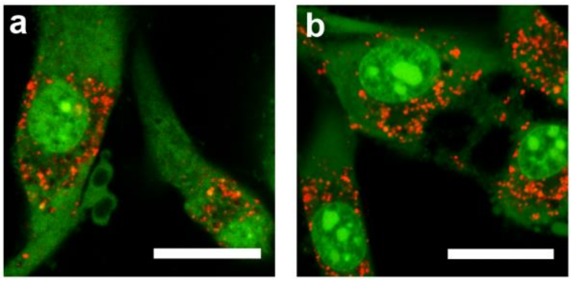

Figure S19. AO-stained images of U87MG cells to observe lysosomal stability after different treatments. (a) Cells were incubated with cRGD-NMe$_2$Br$_2$BDP NP and irradiated with 808 nm laser for 300 s.(b) Cells were incubated with cRGD-NEt$_2$Br$_2$BDP NP and vitamin C, and irradiated with 808 nm laser for 300 s. Scale bars: 20 μm.

6. Supplemental References

(S1) C. Zhan, B. Gu, C. Xie, J. Li, Y. Liu and W. Lu, *J. Control. Release*, 2010, **143**, 136–142.

(S2) A. Gorman, J. Killoran, C. O'Shea, T. Kenna, W. M. Gallagher and D. F. O'Shea, *J. Am. Chem. Soc.*, 2004, **126**, 10619–10631.

(S3) G. A. Crosby and J. N. Demas, *J. Phys. Chem*., 1971, **75**, 991–1024.

(S4) J. Tian, L. Ding, H. J. Xu, Z. Shen, H. Ju, L. Jia, L. Bao and J. S. Yu, *J. Am. Chem. Soc.*, 2013, **135**, 18850–18858.

(S5) N. Adarsh, R. R. Avirah and D. Ramaiah, *Org. Lett.*, 2010, **12**, 5720–5723.

(S6) W. Spiller, H. Kliesch, D. Wöhrle, S. Hackbarth, B. Röder and G. Schnurpfeil, *J. Porphyr. Phthalocya*., 1998, **2**, 145–158.
